# *Chlorella vulgaris* production enhancement with supplementation of synthetic medium in dairy manure wastewater

**DOI:** 10.1186/s13568-016-0184-1

**Published:** 2016-02-20

**Authors:** Jun Shi, Pramod K. Pandey, Annaliese K. Franz, Huiping Deng, Richard Jeannotte

**Affiliations:** State Key Laboratory of Pollution Control and Resources Reuse, College of Environmental Science and Engineering, Tongji University, Shanghai, 200092 People’s Republic of China; Department of Population Health and Reproduction, School of Veterinary Medicine, University of California-Davis, 1089 Veterinary Medicine Drive, Davis, CA 95616 USA; University of California Cooperative Extension, Davis, CA 95616 USA; Department of Chemistry, University of California-Davis, Davis, CA 95616 USA; Universidad de Tarapacá, Avenida General Velásquez No. 1775, Arica, Chile; Department of Plant Sciences, University of California-Davis, Davis, CA 95616 USA

**Keywords:** Dairy wastewater, Algae biomass, Fecal bacteria, Treatment, TEM

## Abstract

To identify innovative ways for better utilizing flushed dairy manure wastewater, we have assessed the effect of dairy manure and supplementation with synthetic medium on the growth of *Chlorella vulgaris*. A series of experiments were carried out to study the impacts of pretreatment of dairy wastewater and the benefits of supplementing dairy manure wastewater with synthetic medium on *C. vulgaris* growth increment and the ultrastructure (chloroplast, starch, lipid, and cell wall) of *C. vulgaris* cells. Results showed that the biomass production of *C. vulgaris* in dairy wastewater can be enhanced by pretreatment and using supplementation with synthetic media. A recipe combining pretreated dairy wastewater (40 %) and synthetic medium (60 %) exhibited an improved growth of *C. vulgaris*. The effects of dairy wastewater on the ultrastructure of *C. vulgaris* cells were distinct compared to that of cells grown in synthetic medium. The *C. vulgaris* growth in both synthetic medium and manure wastewater without supplementing synthetic medium was lower than the growth in dairy manure supplemented with synthetic medium. We anticipate that the results of this study will help in deriving an enhanced method of coupling nutrient-rich dairy manure wastewater for biofuel production.

## Introduction

The dairy industry in the USA has greater than 9 million dairy cows and produces more than 20 million tons of manure annually (Smith et al. 2015). The use of dairy manure wastewater (DMW) for value-added products has the potential to enhance the income of the dairy industry substantially. Only a fraction (<4 %) of the dairy manure produced in the USA is utilized by anaerobic digesters for producing biogas (renewable energy) (USEPA 2014). Therefore, the exploitation of nutrient rich DMW for algal biomass production and phyco-remediation is an option for enhancing the sustainability of dairy industries as well as controlling the adverse impacts of DMW to the environment and to public health (Cantrell et al. 2008). The nutrients of dairy manure can be utilized to cultivate various strains of microalgae with applications for biofuel feedstock; however, considerable difficulties exist for fully exploiting the use of dairy manure wastewater due to its complex nature (i.e., relatively high level of solids and fibers).

Previous studies have shown that various forms of dairy, piggery, municipal, digested, and undigested wastes can be utilized for cultivating algal biomass and enhancing energy production (Chiu et al. 2015; Ding et al. 2014; Kesaano and Sims 2014; Mandal and Mallick 2011; Mulbry and Wilkie 2001; Mulbry et al. 2008; Qin et al. 2014; Wang et al. 2010; Wilkie and Mulbry 2002; Anthony et al. 2015; Hena et al. 2015; Kothari et al. 2013; Kumar et al. 2010; Passero et al. 2015; Zhou et al. 2014; Johnson and Wen 2010). Many of previous studies (Mulbry and Wilkie 2001; Mulbry et al. 2008; Qin et al. 2014; Wang et al. 2010; Wilkie and Mulbry 2002) used anaerobically digested dairy manure for assessing the growth of algae. As an example, Mulbry and Wilkie (2001) proposed the use of dairy manure for growing benthic freshwater algae as a potential alternative to land application of livestock manure for crop production. A study by Kothari et al. (2013) described cultivation of *Chlamydomonas polypyrenoideum* integrated with phyco-remediation of dairy wastewater showing 50–90 % pollution reduction. Wang et al. (2010) tested a semi-continuous *C. vulgaris* cultivation system for treating diluted (×20) digested and undigested dairy manure showing 50–90 % reductions of carbon, nitrogen, and phosphorous. Similarly, Wilkie and Mulbry (2002) showed that higher dairy wastewater nitrogen loading rates resulted in elevated nitrogen content in algal biomass. The authors also inferred that nitrogen uptake of 1430 kg/ha/year can be obtained by growing benthic freshwater algae using dairy wastewater with only 26 % of the required land area that would be needed for using the equivalent nitrogen in a traditional cropping system. However, another study by Mulbry et al. (2008) showed no consistent relationship between loading rate, type of manure, supplement CO_2_ supply and algal biomass fatty acid content (fatty acid content was 0.6–1.5 % of dry biomass weight). Mandal and Mallick (2011) described the cultivation of *Scenedesmus obliquus* for biomass production using poultry litter and municipal secondary settling tank discharges. The authors reported that biomass production was enhanced when these wastes were mixed, and the growth was significantly greater than that in a synthetic media (N 11) (Mandal and Mallick 2011). A study by Ding et al. (2014) described cultivation of microalgae in dairy wastewater and reported that dilution of dairy wastewater increases the nitrogen and carbon removal by microalgae. Despite the considerable research interest in algal biomass production and exploring various concepts, substantial limitations in algal culturing exist related to exploiting the use of various waste sources for algal biomass production (Passero et al. 2015; Gentili 2014; Vardon et al. 2011).

In order to utilize various wastewaters for algae biomass production, pretreatment of the waste stream seems to be inevitable (Passero et al. 2015; Passos et al. 2014). Pretreatments such as hydrothermal (Passero et al. 2015), microwave (Passos et al. 2014), and UV irradiation (Kesaano and Sims 2014; Qin et al. 2014) have been proposed for treating various waste streams, including dairy manure wastewater, to enhance algal biomass production; however, additional understanding is still needed to make pretreatment a viable solution for algae production at a large scale. Enhancing the understanding of how various forms of dairy wastewater, with and without pretreatments, can be utilized still requires further exploration. In addition, dairy wastewater contains elevated levels of animal waste borne bacteria compared to other wastewater (Pandey and Soupir 2011; Pandey et al. 2015) and greater understanding of the effects of animal waste-borne bacteria on algal growth is needed. Pretreatment methods such as waste sterilization and centrifugation can reduce the bacterial biomass in dairy waste substantially; however, it is not clear how these processes affect algal growth and biomass productivity.

The goal of this study is to assess the cultivation of *Chlorella vulgaris* using flushed dairy manure wastewater (FDMW) as an alternate source of dairy waste. While previous studies have explored the use of various forms of dairy wastes and the effluent of anaerobic digesters for algae cultivation (Ding et al. 2014; Kumar et al. 2010; Anthony et al. 2015), the use of FDMW has not been explored previously. While some previous studies have utilized flushed dairy manure (Mulbry and Wilkie 2001; Wilkie and Mulbry 2002), the flushed wastewater was treated by anaerobic digesters before using it for algae production. In existing practices, FDMW from dairy barns is passed through a solid separator to remove manure solids and the remaining liquid fraction is stored in lagoons. Subsequently, both the nutrient-rich manure solids and liquid fraction can be applied onto cropland as fertilizers. Therefore, the exploitation of nutrient rich FDMW for cultivating algal biomass is an important option for enhancing the sustainability of dairy industries as well as controlling the adverse impacts of FDMW to the environment and to public health (Cantrell et al. 2008; Wilkie and Mulbry 2002). This study also compares the impact of two treatment methods (centrifugation and sterilization) of dairy wastewater and the effect of supplementation with synthetic medium (SM). The growth of *C. vulgaris* in raw flushed dairy manure wastewater (RFDMW) (i.e., centrifuged) and sterilized flushed dairy manure wastewater (SFDMW) was compared to understand the impacts of fecal borne bacteria on algal biomass production. Transmission electron microscopy (TEM) of algal biomass grown in RFDMW, SM, and SFDMW is used to understand the ultrastructure of *C. vulgaris* under different growth conditions. Additionally, we have compared the growth of *C. vulgaris* in FDMW obtained from three different dairies to understand the potential changes in algal biomass productivity with changing sources of dairy manure wastewater.

## Materials and methods

### RFDMW, SFDMW, and SM

FDMW was collected from three manure storage lagoons located in three different dairy farms in Merced, Glenn, and Tulare Counties of California, USA. These dairy farms house ≈3000–5000 dairy cows including both milking and non-milking cows. In these dairy farms, the FDMW passes through a solid separator before entering into the lagoon. Once collected from the lagoon, the FDMW was stored at 4 °C prior to starting the experiment. The FDMW average total nitrogen (TN), total solid, carbon, and pH were 2950 (± 429) mg/L, 1.27 (± 0.74) %, 0.36 (± 0.20) %, and 7.7 (± 0.05), respectively. Total phosphorous (TP) of FDMW in wastewater of similar lagoons in the same regions are reported to vary from 141 to 3263 mg/L (with median of 972 mg/L) (Pettygrove 2010). The FDMW was centrifuged (ThermoFisher Sci.: Sorvall Legend X1R) at 10,000 rpm for 15 min. Subsequently, the supernatant was used as RFDMW feedstock for growing *C. vulgaris*. The TN and TP of initial RFDMW were 156.4 and 12.7 mg/L, respectively. RFDMW was sterilized at 121 °C for 15 min to inactivate manure-borne microbial population, and this sterilized manure was used as SFDMW feedstock for growing *C. vulgaris*. The TN and TP of initial SFDMW were 56.6 and 12.7 mg/L, respectively. Established procedures (APHA 1999) were used for observing TN and TP.

To test the effect of supplementation with SM on *C. vulgaris*, we used a blue-green medium (BG-11), a recipe commonly used for growing freshwater algae including *C. vulgaris* (FACC 2014; UTEX 2014). The BG-11 (i.e., SM) was prepared by mixing 958 mL of distilled water, NaNO_3_ (0.25 g), K_2_HPO_4_·3H_2_O (0.075 g), MgSO_4_·7H_2_O (0.075 g), CaCl_2_·2H_2_O (0.025 g), KH_2_PO_4_ (0.175 g), NaCl (0.025 g), 40 mL of soil extract solution, FeCl_3_·6H_2_O (0.005 g), 1.0 mL of Fe-EDTA solution, and 1.0 mL of A5 solution. The SM was autoclaved and stored in 4 °C before using it for growing *C. vulgaris.* To prepare soil extract solution for mixing into SM, we used 200 g unfertilized garden soil and 1000 mL distilled water, heating in a water bath (at 100 °C) for 3 h, and then cooling for 24 h. Then the solution was filtered (0.45 µm) and supernatant was used as a soil extract solution. The Fe-EDTA solution was prepared by mixing 50 mL distilled water, Na_2_EDTA (1.0 g), FeCl_3_·6H_2_O (81 mg) and 0.1 N HCl (50 mL). The composition of the A5 solution was H_3_BO_3_ (2.86 g/L), MnCl_2_·4H_2_O (1.86 g/L), ZnSO_4_·7H_2_O (0.22 g/L), Na_2_MoO_4_·2H_2_O (0.39 g/L), CuSO_4_·5H_2_O (0.08 g/L) and Co(NO_3_)_2_·6H_2_O (0.05 g/L).

### Experiment design

The growth of *C. vulgaris* was assessed in RFDMW, SFDMW, and SM using 500 mL conical flasks under controlled temperature conditions (25 ± 1 °C). The strain of *C. vulgaris* (UTEX-2714) was obtained from the culture collection of algae, University of Texas, Austin, USA. The pre-cultured *C. vulgaris* (OD 680 ≈ 0.355) was inoculated into a 300 mL volume of medium (in 500 mL conical flasks) with a proportion of 20 % (v/v) under sterile conditions. In order to avoid the potential ambient contamination, the experiments were conducted in a biological controlled environment (i.e., inside a bio-safety cabinet level II (SterilGARD Hood, Baker Company)). The bio-safety cabinet was converted into a photo-bioreactor by equipping it with controlled light (two 4 ft. T12 40-w Cool White Supreme (4100 K) Alto Linear Fluorescent Light Bulb with brightness of 2600 lumens) and a temperature control facility. The temperature of bio-reactor was controlled using a heating/cooling tower (Dyson-AM09 Fan, Model: 302198-01) equipped with a sensor for controlling heating and cooling precisely. The growth of *C. vulgaris* in RFDMW, SFDMW, and SM was monitored over 10 days at 25 ± 1 °C. The growth experiment was conducted in dark (12 h) and light (12 h) cycle conditions using 300 mL of growth media in 500 mL conical flasks. The experiment was continued to 30 days, but no increase in cell density was observed beyond 10 days. Previous studies have used a similar growth period of 10 days for assessing the growth of algal biomass in various wastewater sources (Hena et al. 2015; Kothari et al. 2013; Passero et al. 2015). The light and dark conditions were controlled using an electric timer (CUTNSTK624, Prime). To mix the growth environment, intermittent shaking was performed twice a day (by hand) for the first 6 days of cultivation.

The growth of *C. vulgaris* in RFDMW and SM was assessed for 10 days. During the 10 day cultivation period, samples of *C. vulgaris* were collected daily for biomass analysis. Biomass analysis was used to compare the growth of *C. vulgaris* in RFDMW and SM. Subsequently, a series of experiments was conducted to determine the effect of supplementing RFDMW with SM. Three mixtures with RFDMW and SM ratio (volumetric basis) of 20:80, 40:60 and 70:30 were used to evaluate the effect of *C. vulgaris* biomass production. To assess the impacts of animal waste-borne microbial population on *C. vulgaris* growth, we compare the growth of *C. vulgaris* in RFDMW and SFDMW in identical growth conditions. Further, a series of experiments (as described previously for RFDMW) was conducted to identify the optimal growth environment for SFDMW feedstock.

### Algal growth and biomass

The growth of *C. vulgaris* was monitored by measuring the OD at a wavelength of 680 nm using previously published approaches (Mulbry and Wilkie 2001; Mulbry et al. 2008; Wang et al. 2010). Colored dissolved organic matter (CDOM) occurs naturally in wastewater because of tannins released from decaying matter. Both CDOM and chlorophyll *a* absorb in the same spectral range, which poses challenges in differentiating absorbance caused by chlorophyll *a* and wastewater. In order to resolve this issue, we have used controls of each level of RFDMW, SFDMW, and SM prior to measurement. First, the OD of these controls were measured and zeroed, and then the OD of actual sample was measured. This process resolved the differentiation issue of CDOM and *C. vulgaris* optical density.

For algal biomass analysis, a 10 mL sample volume was centrifuged at 8000 rpm for 10 min, and the centrifuged pellets were washed twice with distilled water to remove the salts and solids. Subsequently, each pellet was resuspended in distilled water and filtered through a 47 mm membrane filter (HAWG047S6, Millipore). The *C. vulgaris* biomass retained in the filter was dried overnight at 60 °C and the final biomass weight was measured. An empirical equation (Eq. 1) was developed (R^2^ = 0.98) for calculating the biomass using OD 680 readings where *BM*_*d*_ is biomass dry weight (g/L).

1 $$ BM_{d} = 0.3386 \cdot OD680.$$

In addition to *BM*_*d*_, volumetric biomass productivity (*P*_*b*_) (g/L/d) and specific growth rate (*µ*) (1/d) were estimated using the reported methods (Blair et al. 2014).

2 $$P_{b} = \frac{{X_{2} - X_{1} }}{{t_{2} - t_{1} }}$$

3 $$\mu = \frac{{\ln \left[ {X_{2} /X_{1} } \right]}}{{t_{2} - t_{1} }}.$$

where, *X*_*1*_ and *X*_*2*_ are the biomass concentration (g/L) on days *t*_*1*_ and *t*_*2*_, respectively.

### Ultrastructure analysis of *C. vulgaris* using TEM

To understand the impacts of RFDMW, SFDMW, and SM on the ultrastructure of *C. vulgaris*, we used TEM analysis. The pellets of *C. vulgaris* were fixed in 2 % paraformaldehyde + 2.5 % glutaraldehyde in 0.1 M sodium phosphate buffer. Subsequently, the pellets were rinsed in buffer and then fixed in 2 % OsO_4_ in the same buffer for 1.5 h. The samples were then dehydrated in a graded series of acetone in PBS (10, 30, 50, 70, and 90 %) for 10 min at each level of acetone. Subsequently, at 100 % acetone, samples were dehydrated for 30 min. A mixture of acetone and resin (1:1) was used for 1 h resin infiltration, which was followed by overnight infiltration with 100 % resin. The next day, fresh resin (100 %) was used for a 2 h infiltration before final embedding and polymerization. Ultrathin sections (50 nm thick) were cut using a Diatome diamond knife and picked up onto copper (carbon coated) grids (200 mesh) then stained with 0.5 % uranyl acetate for 2 h and 3 % lead citrate for 5 min before viewing in a Philips CM120 electron microscope. An accelerating voltage of 80 kV and magnification of 13.90 kx were used for examining the specimen.

## Results

### Growth of *C. vulgaris* in RFDMW and SM

**Fig. 1:**
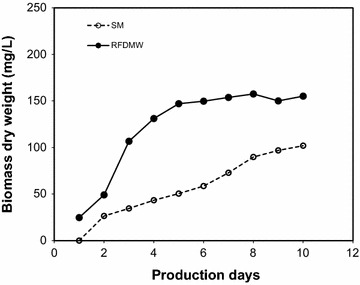
Comparison of *C. vulgaris* growth in RFDMW and SM

Figure 1 shows the growth of *C. vulgaris* in RFDMW and SM indicating the considerable higher biomass dry weight obtained with RFDMW compared to SM. As shown in Fig. 1, the growth of *C. vulgaris* in SM remained steady while a spike in the growth of *C. vulgaris* was observed after 2 days of cultivation in RFDMW. The initial biomass of *C. vulgaris* in RFDMW (24.7 mg/L) and SM (25.4 mg/L) was comparable. At the end of 10 days cultivation time, *C. vulgaris* biomass weight in RFDMW was 155.1 mg/L, while biomass weight in SM was 102.0 mg/L, about 34 % less than the RFDMW.

### Supplementation of RFDMW with SM

**Fig. 2:**
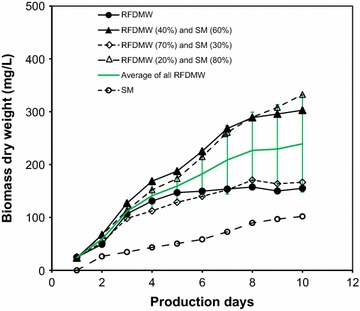
Comparison of *C. vulgaris* growth using various RFDMW/SM ratios. *Solid green line* with *error bars* indicate the average of growth in all RFDMW and SM mixtures

In order to determine the effects of supplementing RFDMW with SM for enhancing the growth of *C. vulgaris*, a comparative growth analysis of *C. vulgaris* was performed using various mixtures of RFDMW and SM (20:80, 40:60 and 70:30). As shown in Fig. 2, the growth of *C. vulgaris* observed using the 20:80 and 40:60 mixtures of RFDMW/SM was higher than the growth in the 70:30 mixture RFDMW/SM, or when using only RFDMW. The average of growth in all RFDMW and SM mixtures is shown as green line (with error bars). On day 1, the average biomass of *C. vulgaris* in RFDMW was 24 mg/L. On day 10 of cultivation, *C. vulgaris* biomass increased to 332.6, 302.8 and 166.6 mg/L, respectively. The production on day 10 at 40:60 mixture (RFDMW/SM) was 48.8 and 66.3 % greater than RFDMW and SM, respectively. Compare to this recipe, the combination of 20:80 (RFDMW/SM) produced slightly better results in raw manure water. As an example, the production on day 10 was 69.3 and 53.4 % greater than that of RFDMW and SM, respectively. The 10 days average biomass dry weight for SM, 20:80, 40:60, 70:30, and RFDMW were 63.9 (±27.8), 193.4 (±105.8), 195.5 (±98.8), 121.0(±49.6), 122.5(±47.9), respectively. These results demonstrate that supplementation of RFDMW with SM can enhance the growth of *C. vulgaris* relative to SM, and a preliminary comparison indicates that both the 40:60 and 20: 80 mixtures of RFDMW/SM are effective ratios. When SM was mixed with RFDMW, the growth was relatively higher than that of SM alone, which indicates that that the RFDMW influences the growth of *C. vulgaris* positively, however, instead of growing *C. vulgaris* in only RFDMW, a recipe combining dairy wastewater with synthetic growth medium can provide optimal conditions for the growth of *C. vulgaris.* The average biomass growth of all mixture of RFDMW is shown in Fig. 2 as solid red line. During 10 days growth period, the average biomass growth of all mixtures of RFDMW varied from 24 (±1.3) to 239.3 (±91.5) mg/L.

### Comparison of *C. vulgaris* growth on SFDMW and SM

**Fig. 3:**
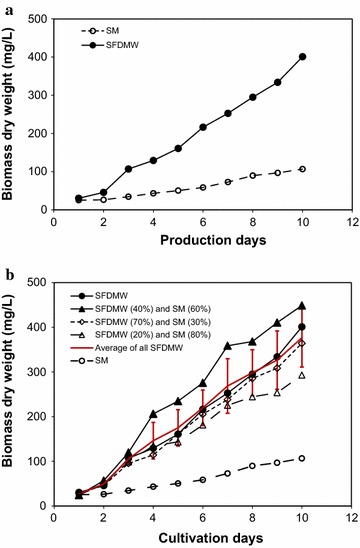
**a** Comparison of *C. vulgaris* growth in SFDMW and SM. **b** Comparison of *C. vulgaris* growth using various SFDMW/SM ratios

Figure 3 shows the growth of *C. vulgaris* in SFDMW compared to SM, and also in mixtures of SM and SFDMW at various ratios (20:80, 40:60 and 70:30). As seen in Fig. 3a, the growth of *C. vulgaris* in SFDMW affords higher biomass dry weight compared to RFDMW and SM. The initial biomass (day 1) of *C. vulgaris* in SFDMW and SM were 30.4 and 25.4 mg/L, respectively, and at the end of 10 days cultivation time, *C. vulgaris* biomass weight in SFDMW reached 401.1 mg/L, while in SM the biomass only reached 101.9 mg/L (approx. 76 % less than SFDMW). As seen in Fig. 3b, the growth of *C. vulgaris* using a mixture of 40:60 SFDMW/SM was relatively higher compared to growth in other SFDMW/SM ratios (i.e., 20:80, 70:30 and 100:0), and all are higher than growth in SM alone. The initial *C. vulgaris* biomass isolated on day 1 was 24.7, 24.4, 29.1 and 30.5 mg/L at ratios of 20:80, 40:60, 70:30 and 100:0, respectively. On day 10 cultivation, *C. vulgaris* biomass levels increased to 293.0, 448.8, 364.1 and 401.0 mg/L, respectively. The average biomass over 10 days for SM was 60.5 (±29.7) mg/L. The values for 20:80, 40:60 and 70:30 (SFDMW/SM) mixes were 164.9 (±90.2), 250.4 (±148.7), and 184.8 (±114.5) mg/L, respectively. Comparison indicates that 40:60 mixture (SFDMW/SM) performed better than other mixtures. The average growth of all SFDMW mixtures (shown in Fig. 3b) varied from 27.2 (± 3.1) to 376.7 (± 65.7) mg/L. The observed higher biomass productivity in both SFDMW and RFDMW compared to only SM further emphasizes the utility of nutrient-rich FDMW for growing *C. vulgaris* (Mulbry and Wilkie 2001; Mulbry et al. 2008).

**Fig. 4:**
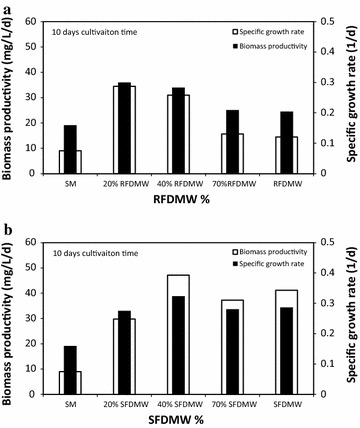
**a** *C. vulgaris* productivity and specific growth rate in SFDMW with 10 days cultivation. **b** *C. vulgaris* productivity and specific growth rate in RFDMW with 10 days cultivation

The overall results suggest that the biomass production of *C. vulgaris* using SFDMW is better than compared to using SM and RFDMW (Fig. 4a, b). Figure 4 shows an initial comparison biomass productivity and specific growth rate of *C. vulgaris* in RFDMW (Fig. 4a) SFDMW (Fig. 4b), and SM under various mixture conditions. As shown (Fig. 4a, b), *C. vulgaris* growth using 40 % SFDMW afforded the highest productivity with a specific growth rate of 0.3 day^−1^. With non-autoclaved condition, the specific growth rate and biomass productivity using 20 and 40 % RFDMW are relatively similar (Fig. 4a), while in autoclaved condition the 40 % RFDMW afforded higher biomass production compared to other mixtures. In 10-day cultivation time, 40 % SFDMW yielded the largest biomass growth of 47.2 mg/L/day, which was 80.8 % greater than the productivity observed using SM (9.0 mg/L/day) and 12.7 % greater than using only SFDMW (41.1 mg/L/day) (Fig. 4b). In 40 % RFDMW, biomass growth (20.6 mg/L/day) was 70.9 and 53.3 % greater than that of SM and RFDMW, respectively. The specific growth rate in 40 % RFDMW (0.28 day^−1^) was 43.6 and 27.9 % greater than SM and RFDMW, respectively. In 40 % SFDMW, specific growth rate of 0.32 (day^−1^) was 50.7 and 11.5 % greater than that of SM and SFDMW.

## Discussion

### Impact of RFDMW on ultrastructure of *C. vulgaris*

**Fig. 5:**
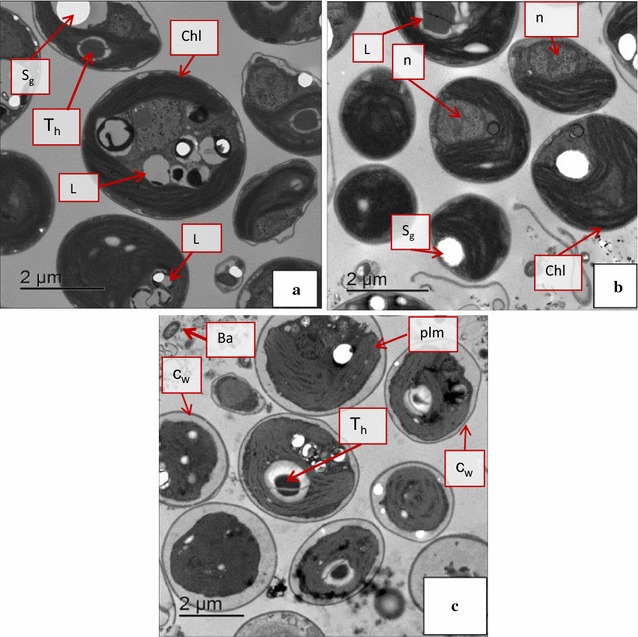
Transmission Electron Micrographs (*Chl* indicates chloroplast; *S*_*g*_ indicates starch granules; *T*_*h*_ shows thylakoid; *C*_*w*_ shows cell wall; *plm* indicates plasma membrane; *n* indicates nucleus; *Ba* indicates bacteria: **a** *C. vulgaris* ultrastructure in SM. **b** *C. vulgaris* ultrastructure in SFDMW. **c** *C. vulgaris* ultrastructure in RFDMW

Using TEM, we compared changes in the ultrastructure of *C. vulgaris* grown in RFDMW, SFDMW and SM conditions (Fig. 5). The use of TEM to observe cell structures of *C. vulgaris* have been reported previously (Yu et al. 2015). Cellular organelles such as the chloroplast, thylakoid, granules, and cell wall are clearly visible using TEM (Fig. 5). The micrographs of *C. vulgaris* cells grown using SM (Fig. 5a), 40 % SFDMW (Fig. 5b), and 40 % RFDMW (Fig. 5c) are shown. Numerous intact *C. vulgaris* algae cells and their lysate were visible for each condition with cell diameters ranging from 1 to 6 µm. Previous studies (Zhao et al. 2014; Yu et al. 2015) used TEM to illustrate lysates inside of the microalgae cells, which influences microalgae lipid yields. The cell lysis (i.e., cell disruption) releases soluble organic compounds including lipids from microalgae cells (Ahn et al. 2002; Huang et al. 2014). Previous studies have shown that increased lipid extraction occurs as a result of cell lysis of microalgae (Ali and Watson 2015; Balasubramanian et al. 2011). Rupturing of cell walls results in oil (i.e., lipids) release from the cells (Mercer and Armenta 2011). The localization of the lipid bodies near the cell walls facilitate their release once the cell walls are ruptured (Cravotto et al. 2008; Wei et al. 2008).

In each TEM (2 µm) we detected nine *C. vulgaris* cells (Fig. 5a, b, c). The cells grown in RFDMW (Fig. 5c) show distinct changes in ultrastructure compared to SFDMW and SM. For cells cultivated using SM or 40 % SFDMW conditions, the entire cell is enclosed by a cell wall and the plasma membrane remains close to the cell wall. However, for cells cultivated in 40 % RFDMW, the plasma membrane was observed to be detached from the cell wall (Fig. 5c). The shrinkage of cytoplasm (with nucleus and membrane) suggests that cell damage is occurring with RFDMW growth conditions. This cell damage was apparent in the micrographs for all cells cultivated in RFDMW medium. Bacterial cells were also observable in RFDMW micrographs (Fig. 5c). Similar cell damage has been observed by Passero et al. (2015) using TEM, when a freshwater algae (*Oocystis* sp.) was grown under thermal pretreatment conditions. Another study by Anthony et al. (2015) utilized TEM to monitor the changes in ultrastructure for *C. vulgaris* over the various growth periods. This study also reported similar intracellular damage upon exposure to UV light. Therefore, there is a common cell damage associated with the cell wall that is observed when microalgae are exposed to UV light, thermal treatment, or dairy wastewater. Notably, these previous studies (Anthony et al. 2015; Passero et al. 2015) also demonstrate that there is a correlation between damaged cell walls in microalgae cells and increased lipid accumulation.

### Comparison of *C. vulgaris* growth using wastewater from different dairy facilities

**Fig. 6:**
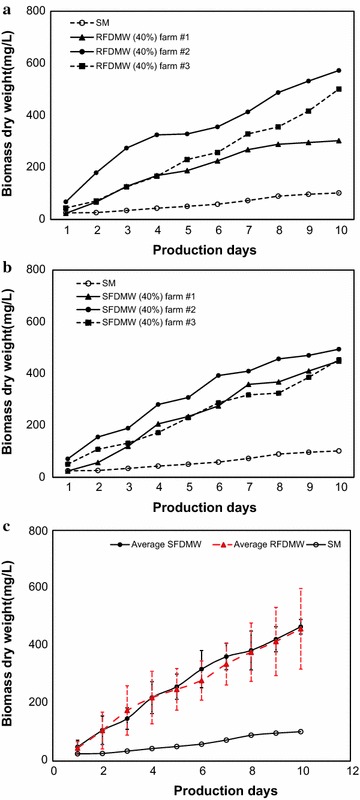
**a** Growth of *C. vulgaris* in SFDMW from three dairy farms. **b** Growth of *C. vulgaris* in RFDMW from three dairy farms (flushed dairy wastewater was collected from three different dairies in three counties (#1: Dairy Farm 1, #2: Dairy Farm 2, and #3: Dairy Farm 3). **c** Comparison of average growth for SFDMW and RFDMW

The growth of *C. vulgaris* was compared using flushed DMW collected from three dairies (including dairy farm #1) with a 40:60 ratio of FDMW/medium for both SFDMW and RFDMW (Fig. 6). When comparing the growth of *C. vulgaris* in 40 % RFDMW, the biomass isolated at the end of the 10 day growth period was 302.7, 572.2, 501.1 mg/L for dairy farm #1,#2 and #3, respectively (Fig. 6a). The average specific growth rate and biomass productivity at 40 % RFDMW were 0.3 day^−1^ and 46.0 mg/L/day. The selection of 40 % SFDMW and 40 % RFDMW for growth of *C. vulgaris* was based on our previous inference that a 40:60 mixture can serve as an optimal combination for increased biomass productivity (vida supra). In 40 % SFDMW, the initial biomass (day 1) of *C. vulgaris* was 24.4, 71.1 and 50.8 mg/L for dairy farm #1, #2 and #3, respectively. At day 10 of the growth period, the biomass increased to 448.6, 494.4, 453.7 mg/L, respectively (Fig. 6b). The average specific growth rate and biomass productivity using 40 % SFDMW is 0.3 day^−1^ and 46.3 mg/L/day, respectively. Consistent with results presented earlier, growth of *C. vulgaris* using SM alone produced considerably lower dry biomass weight compared to all FDMW from all three dairy farms.

The average results for all three dairies with both RFDMW and SFDMW (Fig. 6c), indicate that the combination of nutrient-rich FDMW with SM provides a better environment for the growth of *C. vulgaris*. A larger variation was observed using RFDMW with the maximum biomass productivity observed using RFDMW from dairy farm #2 and the minimum biomass productivity observed using RFDMW from dairy farm #1. Nevertheless, all three tests showed that the growth of *C. vulgaris* was improved when FMDW was mixed with SM (Fig. 6). While the SFDMW and RFDMW conditions demonstrated the same average biomass production, the pretreatment of FDMW was effective to reduce variations resulting from different dairies, such as different levels of animal waste-borne bacteria.

## Abbreviations

DMW: dairy manure wastewater
FDMW: flushed dairy manure wastewater
RFDMW: raw flushed dairy manure wastewater
SFDMW: sterilized flushed dairy manure wastewater
SM: synthetic medium
TEM: transmission electron microscopy
TN: total nitrogen
TP: total phosphorous
*C. vulgaris*: *Chlorella vulgaris*
